# Barriers and facilitators to opioid agonist therapy in rural and remote communities in Canada: an integrative review

**DOI:** 10.1186/s13011-022-00463-5

**Published:** 2022-08-26

**Authors:** Em M. Pijl, Abeer Alraja, Elsie Duff, Carol Cooke, Stephen Dash, Nichole Nayak, Jesse Lamoureux, Ginette Poulin, Erin Knight, Ben Fry

**Affiliations:** 1 Present Address: University of Manitoba, Winnipeg, Canada; 2 Pine Falls Health Centre, Powerview-Pine Falls, Canada; 3 Rapid Access to Addictions Medicine, Winnipeg, Canada; 4 Health Science Centre Winnipeg, Winnipeg, Canada; 5 Shared Health Manitoba, Winnipeg, Canada

**Keywords:** Rural population, Opioid epidemic, Opioid agonist, Opioid-related disorders, Community health services, Integrative review

## Abstract

**Background:**

People living in rural and remote communities in Canada are often disproportionately impacted by opioid use disorder. When compared to urban centres, rural and remote populations face additional barriers to treatment, including geographical distance as well as chronic shortages of health care professionals. This integrative review of the literature was conducted to explore the facilitators and barriers of OAT in rural and remote Canadian communities.

**Methods:**

A search of the literature identified relevant studies published between 2001 and 2021.

**Results:**

The search strategy yielded 26 scholarly peer-reviewed publications, which explored specific barriers and facilitators to rural and remote OAT in Canada, along with two reports and one fact sheet from the grey literature. Most of the scholarly articles were descriptive studies (*n* = 14) or commentaries (*n* = 9); there were only three intervention studies. Facilitators and barriers to OAT programs were organized into six themes: intrapersonal/patient factors, social/non-medical program factors, family/social context factors (including community factors), infrastructure/environmental factors, health care provider factors, and system/policy factors.

**Conclusions:**

Although themes in the literature resembled the social-ecological framework, most of the studies focused on the patient-provider dyad. Two of the most compelling studies focused on community factors that positively impacted OAT success and highlighted a holistic approach to care, nested in a community-based holistic model. Further research is required to foster OAT programs in rural and remote communities.

Opioid agonist therapy (OAT) is widely considered the gold standard treatment for opioid use disorder [1, 2]. While the accessibility of OAT has improved in some Canadian jurisdictions, its use and availability continue to lag in rural and remote communities [3]. This lag is concerning because rural and remote populations are often disproportionately impacted by opioid use disorder and face additional barriers to treatment when compared to urban populations [3]. In addition, rural and remote OAT services across Canada can vary widely in their quality and breadth, where some communities receive highly innovative care and others receive relatively ineffectual care [4, 5]. Inconsistent or ineffectual OAT programs can result in high rates of patient attrition, which can have a devastating impact on individuals, families, and communities [5]. The purpose of this integrative review was to explore the facilitators and barriers of OAT in rural and remote Canadian communities. The findings of this review can provide a foundation for researchers, policy makers, and knowledge users to develop a shared research and practice agenda.

## Background

Rural and remote communities in Canada have been disproportionately impacted by opioid use disorder (OUD) [6] and the current opioid epidemic. People in these communities experience additional barriers to treatment, including geographical factors and chronic shortages of health care professionals, when compared to their urban peers [7–10]. In addition, OAT programs in these communities can be ineffectual or inconsistent with high rates of patient attrition.

OAT is considered the gold standard for patients with OUD. In OAT, patients substitute problematic illicit and extra-medical opioids (such as oxycodone and fentanyl) with an ongoing prescription for buprenorphine (with or without naloxone) or methadone, both of which are long-acting opioids that stimulate receptors in the brain to prevent withdrawal and reduce cravings [11]. OAT is usually combined with in-clinic visits and daily witnessed ingestion of the medications at a pharmacy.

OAT can positively impact patients, families, and communities by reducing substance use, increasing family stability, decreasing crime rates, improving mental and physical health, and enhancing quality of life [6, 12]. Consistent and prolonged engagement in OAT is associated with lower morbidity and mortality as well as higher quality of life for patients seeking to reduce or cease opioid use [13, 14].

Retention of patients on OAT can be complicated due to a wide range of intrinsic, extrinsic, and environmental factors, such as shortages of health care personnel, lack of technology for telehealth, failure of medication doses to meet the patient’s needs, continued illicit drug use, and lack of social supports [8, 15–19]. Unfortunately, the premature cessation of OAT is associated with a variety of negative outcomes [20–22], including drug overdose, bloodborne infections, worsening substance use, and death [19, 23–25].

People in rural and remote communities have higher rates of OUD and experience greater difficulty accessing help when compared to their urban counterparts [19, 26]. Patients in rural and remote locations face barriers to OAT, including geographical distances to clinics and pharmacies, limited seasonal accessibility, chronic shortages of health care professionals, and poor access to complementary supports such as psychotherapy [7–9, 27]. Consistent with these findings, Rush and Furlong [28] advocated that people in rural and remote regions of Canada need better access to substance use care than is currently provided. Given the significant increase in opioid use that has arisen during the COVID-19 pandemic [29], this matter is both timely and urgent.

While best practices for OAT have been outlined by the Canadian Centre on Substance Use and Addiction [30], recommendations specific to rural and remote populations are still lacking. Nevertheless, some research regarding rural and remote OAT has been published in recent years. For instance, Venner et al. [31] reported on a key stakeholders’ meeting about the acceptability of OAT among Indigenous people in the United States, elucidating a considerable gap between Western medical perspectives/approaches (including OAT) and traditional/Indigenous healing approaches [32]. The First Nations Health Authority [33] articulated some practical tips for rural and remote communities, such as: increasing the role of community health facilities; expanding the role of registered nurses in OAT; and, ensuring the availability of wraparound supports, including peer support, case management, and culturally relevant healing practices. Levine et al. [32] described an Indigenized approach to harm reduction that is both holistic and culturally safe. These are just a few examples of recommendations that need to be synthesized in the forefront of the scholarly literature and translated into practice settings.

## Methods

The objective of this review was to explore the facilitators and barriers of outpatient OAT in rural and remote Canadian communities (some of which are Indigenous communities). Integrative reviews aim to address a research problem through compiling evidence from multiple study designs and types of data in order to more fully understand a phenomenon of concern [34, 35]. Integrative reviews explore how concepts are described in the literature, what work has been done and needs to be expanded, what relationships have been explored between concepts or other related phenomena, and what research approaches have been used to study the concept [34].

This integrative review was conducted according to the guidelines put forth by the Joanna Briggs Institute [36] and leading methodologists [34, 35]. The search terms were developed by the principal investigator with a health sciences librarian. The search terms included OAT (and its variant terms) medications as subject headings, key words describing geographic locations (rural, remote, and variants), and key words pertaining to Indigenous communities, since many rural and remote communities in Manitoba are predominantly Indigenous. Search terms for medications were limited to opioid substitution therapies; as a result, heroin, hydromorphone, and other opiates were not included in the search, as these drugs are associated with safe supply rather than substitution. A pilot search was conducted to refine the search terms and assess the final search results against a list of exemplar articles.

The literature search aimed to identify relevant studies published between 2001 and 2021, using MEDLINE (see Table 1), EMBASE, CINAHL, Cochrane Library, PubMed, Health and Psychosocial Instruments, Scopus, JBI Databases of Systematic Reviews and Implementation Reports, Cochrane, ERIC, Web of Science, Google Scholar, ScienceDirect and PsycINFO. Hand searches were conducted to identify grey literature in trial databases (Canadian Electronic Library, Health Services Research Projects in Progress, Canadian Institutional Repositories, ProQuest Dissertations and Theses) as well as on websites of relevant organisations (e.g. CRISM, BCCSU, CADTH), provincial and territorial health ministries, and federal health bodies (Health Canada, PHAC). Articles were included in the review if they met the following criteria: represented original research (qualitative, quantitative, and mixed methods as well as theses and dissertations) about OAT; written in English (due to lack of available translator[s]); based in Canada; and addressed a rural or remote issue pertaining to OAT. Literature was not limited to adults because OAT is also useful among pediatric patients.

**Table 1:** Ovid Medline Search Strategy

Line	Concept/KW/SH
1	methadone.mp
2	exp Methadone/
3	exp Buprenorphine/
4	exp Buprenorphine, Naloxone Drug Combination/
5	buprenorphine.mp
6	buprenorphine-suboxone.mp
7	exp Methadyl Acetate/
8	Methadyl Acetate.mp
9	"methadone maintenance".mp
10	"methadone maintenance therapy".mp
11	"methadone maintenance treatment".mp
12	"methadone substitution".mp
13	"buprenorphine maintenance therapy".mp
14	exp Opiate Substitution Treatment/
15	"opiate substitution treatment".mp
16	"opioid substitution therapy".mp
17	exp Narcotic Antagonists/
18	"opioid agonist therapy".mp
19	"opioid agonist treatment".mp
20	"opiate substitution".mp
21	"opiate substitute".mp
22	"opioid substitute".mp
23	"opioid replacement".mp
24	"medications for opioid use disorder".mp
25	1 or 2 or 3 or 4 or 5 or 6 or 7 or 8 or 9 or 10 or 11 or 12 or 13 or 14 or 15 or 16 or 17 or 18 or 19 or 20 or 21 or 22 or 23 or 24
26	rural.mp
27	Rural Health/
28	Rural Health Services/
29	Rural Population/
30	remote.mp
31	regional.mp
32	Health Services, Indigenous/
33	aborigin*.mp
34	exp Indigenous Peoples/
35	indigenous.mp
36	exp Indigenous Canadians/
37	Inuits/
38	inuit.mp
39	exp Indians, North American/
40	metis.mp
41	"first nation".mp
42	"first nations".mp
43	"native people".mp
44	"native peoples".mp
45	amerindian.mp
46	exp American Natives/
47	"native american".mp
48	"native americans".mp
49	exp Alaskan Natives/
50	"alaska* native".mp
51	American Native Continental Ancestry Group/
52	26 or 27 or 28 or 29 or 30 or 31 or 32 or 33 or 34 or 35 or 36 or 37 or 38 or 39 or 40 or 41 or 42 or 43 or 44 or 45 or 46 or 47 or 48 or 49 or 50 or 51
53	25 and 52
54	exp Canada/
55	(british columbia or alberta or saskatchewan or manitoba or ontario or quebec or new brunswick or nova scotia or prince Edward island or newfoundland or labrador or nunavut or nwt or northwest territories or yukon or nunavik or Inuvialuit).mp. [mp = title, abstract, original title, name of substance word, subject heading word, floating sub-heading word, keyword heading word, organism supplementary concept word, protocol supplementary concept word, rare disease supplementary concept word, unique identifier, synonyms]
56	canad*.mp
57	54 or 55 or 56
58	53 and 57
59	north america/ or exp united states/ or exp Canada/
60	53 and 59
61	55 or 56 or 59
62	53 and 61
63	limit 62 to english language
64	limit 63 to yr = "2001—2022"

Full texts were pulled by research assistants and Covidence [37] was used as an organizing and collaborating tool. The principal investigator and three research assistants independently reviewed titles and abstracts to assess for inclusion criteria related to the population, concept, and context as well as to remove duplicates. This process was followed by a more thorough reading to assess for inclusion. Any disagreements between team members regarding the inclusion of specific articles were discussed and resolved within the team [38].

Researchers recorded the following information for each article: author(s); year of publication; type of article (academic or grey); country of origin (in case the search needed to be expanded beyond Canadian literature); aim/purpose; study population and sample size; methodology/method; intervention type/duration and outcome measures; and key findings, specifically related to facilitators and barriers to OAT. Since this integrative review focused on exploring the facilitators and barriers of OAT in rural and remote Canadian communities, researchers commented on the quality of each article but did not omit less robust items, as would be done in a systematic review. Thus, this review was focused on relevance rather than quality.

While definitions of “urban”, “rural” and “remote” vary widely by source and application and there is no consensus on definitions [39], the Statistics Canada definition of “rural” was adopted for the purposes of this research. Statistics Canada differentiates between rural and population centres (formally “urban” centres) as follows. A population centre is defined as having a population of over 1,000 people and a density of 400 people per square kilometre. If an area does not meet these criteria, it is considered rural. Additionally, population centres are divided into small, medium and large, with small population centres having a population of 1,000 to 29,999 and a density of 400 people per square kilometre. Similarly, there is little consensus on what constitutes “remote” in the literature and the term is used to describe a range of communities that are significant distances from population centres and that lack access to services [40]. We included articles that self-declared their communities to be “rural” or “remote” and then followed up with Statistics Canada to ensure the threshold of rurality was not exceeded.

## Results

The search strategy returned 230 records from all databases. Fifty-eight duplicate records were removed and 172 titles and abstracts were screened (excluding 123 references). Of the remaining 49 full-text citations, 29 references met all inclusion criteria. These references included 26 scholarly peer-reviewed publications, which explored specific barriers and facilitators to rural and remote OAT in Canada (see Table 2), and were comprised of 14 research studies, nine commentaries, one case report, and two review articles. Most of the studies were conducted in Ontario (*n* = 21) and British Columbia (*n* = 2).

**Table 2:** Studies on facilitators and barriers to access to and retention on OAT in rural, remote and Indigenous Canadian communities

**Authors**	**Location**	**Rurality (Statistics Canada, 2020) [**39**]**		**Type**	**Findings**
		**Population**	**Density**		
**Peer-Reviewed**					
Bardwell and Lappalainen (2021) [41]	Canada	Non-specific rural	N/A	Commentary	There are gaps in research and policy directives for safer supply and novel OAT programs in smaller settings. Smaller communities should explore virtual OAT and safer supply clinics, pharmacist or nurse-led OAT and safer supply home delivery services to overcome barriers to access. The involvement and inclusion of PWUD in the development and delivery of programs is necessary to create more effective programming.
Buck-McFadyen et al. (2020) [42]	ON	4065	17.9/km^2^	Commentary	The Rural Outpatient Opioid Treatment program provides access to an interdisciplinary team, opioid agonist therapy, counselling, and peer support for people experiencing opioid use problems. The ROOT program provides wrap-around services with integrated medical, social, and peer supports.
Dorman et al. (2018) [43]	ON	FN community; no data in Statistics Canada	No data	Commentary	A community working group can strengthen relationships and create a culturally relevant program. Investing in community-based opioid dependence treatment programs that incorporate cultural and land-based healing strategies and draw on First Nations teachings is essential for treating OUD in First Nations communities. There is a need for cultural-based treatment modalities that are based on collaboration between a local hospital, community members, and First Nations.
Eibl et al. (2016) [44]	ON	Non-specific rural	N/A	Commentary	There is a general lack of services in rural and remote communities. OAT and other harm reduction strategies should be available to all opioid-dependent people as first-line treatments. OAT is the best practice for long term patient safety, social stabilization, and long-term health benefits. More resources (including more prescribers) are needed to enable comprehensive care to improve health outcomes.
Jumah et al. (2018) [45]	ON	5839	15.4/km^2^	Commentary	Highlights the outcomes of workshops for health care providers of substance-involved pregnant and parenting women. Participants identified the need for improved transitions in care, facilitated access to buprenorphine treatment, improving postpartum care for mother and child, stable funding models for addiction programs and a focus on Indigenous-led programming. There is a need for a national strategy to address the effects of opioid use in pregnancy from a culturally safe, trauma-informed perspective.
Uddin (2013) [46]	ON	977	4.1/km^2^	Commentary	Programs need to be developed by the people from the communities they serve. Community ownership of the program improves success rates. Support programs must incorporate Indigenous cultural values to ensure the program’s success and meet the needs of the participants. Prescription drug addiction programs must include healing that focuses on medicine wheel teachings (physical, mental, spiritual, and emotional well-being).
Webster (2013) [47]	ON	108,243 and surrounding rural communities	332.1/km^2^	Commentary	The over-prescribing of opioids by physicians working on short-term contracts for Health Canada caused widespread addiction in many First Nations communities. The prescription opioid problem added more suffering for communities already experiencing poverty, unemployment, inadequate housing, polluted drinking water, and high suicide rates. The Government of Canada’s response to address OUD in First nations communities is slow.
Wendt et al. (2021) [48]	North American, Canada	Non-specific First Nations communities	N/A	Commentary	The COVID-19 pandemic intensified opioid use problems within Indigenous communities. Increasing take-home carries for patients has created better access to OAT, lowered stigma, and promoted greater self-efficacy for patients. Indigenous-serving clinics have expanded telemedicine services, giving better access to treatment. The pandemic has limited the ability to participate in traditional Indigenous healing practices which are important for cultural connectedness and recovery.
Weng (2020) [49]	BC	Non-specific rural	N/A	Commentary	A province-wide, centralized virtual care program for patients in rural and remote areas to access OAT can help combat the opioid overdose crisis. Telemedicine can reduce barriers to OAT and enhance retention for both OAT and addictions counseling.
Jones and Quinn (2020) [50]	NWT	Non-specified remote		Case Report	Buprenorphine/naloxone might not be regularly stocked in rural pharmacies, and business hours may be limited. Nurses may be unfamiliar with OAT in remote settings. More education and acceptance of OAT prescribing is required for ongoing OAT. The safety profile of buprenorphine enables more liberal dispensing if a patient is living in a remote community.
Franklyn et al. (2016) [3]	ON	Non-specific rural, remote and First Nations	N/A	Review	In Northern Ontario, OAT success is impacted by geography, treatment modality, and concurrent polysubstance use among OAT patients. Rural and remote communities can benefit from alternative modes of care, such as telemedicine. Better infrastructure is needed, as physicians in these communities need authorization to prescribe OAT, and mentorship from addiction and pain specialists. Benzodiazepine use and cocaine use among OAT patients can often correlate with early dropout from OAT programming.
Jumah et al. (2015) [10]	ON	Non-specific rural, remote and First Nations	N/A	Literature review	Indigenous Canadians are disproportionately affected by opioid misuse. Methadone maintenance therapy (MMT) has logistical limitations in rural and remote settings, but buprenorphine and slow-release Kadian maintenance therapies are feasible alternatives. Regulatory changes are needed to enhance access treatment for pregnant women to access OAT treatments during pregnancy and after giving birth. Post-partum treatment for opioid dependency is often not provided to women living in rural or remote communities, therefore, better access to OAT could help prevent relapse and involvement with child and family services.
Dooley et al. (2018) [51]	ON	5839	15/4/km^2^	Research, Descriptive	Pregnant females in treatment with buprenorphine-naloxone had better pregnancy outcomes than females with illicit opioid use and similar outcomes to females who were not using opioids during pregnancy. A retrospective chart review at the Sioux Lookout Meno Ya Win Health Centre catchment area indicates that lower rates of neonatal abstinence syndrome may be due to rural community-based prenatal, OAT and addictions services.
Eibl et al. (2015) [27]	ON	Various rural, remote and First Nations; rural defined by Rurality Index of Ontario	Unspecified	Research, Descriptive	An observational cohort study using administrative health care databases for patients who began methadone maintenace therapy revealed regional differences in retention rates and mortality of first-time MMT. Patients with limited access or geographical barriers to OAT treatment experience higher retention rates when they access therapy. Self-motivation to seek help and access treatment is likely an important factor in the higher retention rates from patients in Northern regions.
Fonseca et al. (2018) [52]	ON	9512, 12,595	14.2/km^2^, 17.7/km^2^	Research, Descriptive	Semi-structured individual interviews with 11 rural pharmacists found that providing OAT at a community pharmacy is associated with increased workload, longer operating hours, challenges hiring staff with OAT training, and concerns about safety. Coordinating MMT services across multiple community pharmacies in the area could help improve access to treatment, as more OAT providers are needed in rural communities.
Franklyn et al. (2017) [53]	ON	Various unspecified rural, remote and First Nations	Unspecified	Research, Descriptive	A retrospective cohort study using anonymized EMR from 58 clinics offering OAT in Ontario found that benzodiazepine use at baseline was predictive of increased attrition. Patients with benzodiazepine-positive urine samples drop out of OAT treatment more often than patients who have no benzodiazepine use. Northern patients who overcome barriers to treatment entry may be more motivated to succeed in treatment.
LaBelle et al. (2018) [7]	ON	Various unspecified rural, remote and First Nations	Unspecified	Research, Descriptive	A retrospective cohort study using an administrative database revealed that telemedicine is increasingly being utilized throughout Ontario for delivering mental health treatment. Similar barriers exist in Northern Ontario between people seeking out psychiatric care and people seeking OAT treatment. OAT can be delivered via telemedicine to increase access to rural patients with OUD, and it is viewed as beneficial for both the patient and provider.
Landry et al. (2016) [54]	NB	2062	134.1/km^2^	Research, Descriptive	A qualitative study using semi-structured focus group discussions with health care professionals, community members and patients found that there are widespread misconceptions about OAT in the community. OAT was associated with improvements in community-level outcomes (e.g. crime reduction). Community education initiatives about OAT can enhance community buy-in and reduce stigma.
Mamakwa et al. (2017) [55]	ON	6 remote communities	N/A	Research, Descriptive	A retrospective cohort study in six First Nations communities in northwestern Ontario explored the interventions of OAT and First Nations healing programming. Treatment retention rates and negative urine drug screen results were higher than those reported for most OAT programs. Success was fostered by community-based programming that incorporates cultural practices and healing circles. Sustainable core funding is needed.
Morin et al. (2021) [29]	ON	Unspecified rural communities	N/A	Research, Descriptive	Electronic medical record (EMR) data from a chain of 67 OAT clinics in Ontario found that the use of fentanyl increased by 108% among OAT patients in Ontario during the COVID-19 pandemic. Reduced monitoring may have decreased OAT effectiveness and negatively impacted patient outcomes. In the future, infection control measures should be implemented rather than reduced monitoring.
Oukachbi and Rizzo (2020) [19]	ON	108,843	332.1/km^2^	Research, Descriptive	Face-to-face interviews using an adapted version of the Addictions Severity Index (ASI) with OAT patients at an outpatient clinic in Thunder Bay. Risk factors for attrition include having a criminal record, using heroin, experiencing a family conflict and living with someone who uses substances. There is a need for individualized, holistic care and integration of support services into OAT programs.
Russell et al. (2019) [56]	ON	52,662 2902 41,145 1200 8057 14,967 7466 7388 5839 108,843 72,051	166.9/km^2^ 0.1/km^2^ 13.9/km^2^ 6.0/km^2^ 95.9/km^2^ 70.7/km^2^ 292.2/km^2^ 112.7/km^2^ 15.4/km^2^ 332.1/km^2^ 324.6/km^2^	Research, Descriptive	A mixed-methods study on treatment barriers for youth who use illicit drugs or misuse prescription drugs. Qualitative analyses from the study found an overall lack of services in the area; barriers to accessing treatment and services included lack of motivation for treatment, stigmatization, long waitlists and transportation/mobility issues. There is a need for harm reduction-based services, low-threshold programs, specialized programming, and peer-based counselling.
Srivastava et al. (2020) [57]	ON	108,843	332.1/km^2^	Research, Descriptive	A survey of high school graduates who were on a school-based OAT program found that offering OAT to youth with OUD in a high school clinic might be an effective strategy for promoting positive long-term health and social outcomes. Retention was associated with recent formal substance use counseling.
Eibl et al. (2017) [58]	ON	Various	N/A	Research, Intervention	This non-randomized cohort comparison study used an administrative database for patients who started OAT via telemedicine, finding that patients treated via telemedicine were more likely to remain in therapy than patients receiving treatment in-person. A significant barrier to telemedicine-delivered OAT treatment is that current Ontario guidelines require physicians to have an in-person appointment with all telemedicine patients within 6 weeks of starting OAT, which can hinder patient retention due to transportation issues.
Kanate et al. (2015) [6]	ON	921	10.3/km^2^	Research, Intervention	Quantitative measurements of community wellness and OAT in a remote First Nations community reveal that one year after the community-developed program of First Nations healing, addiction treatment, and OAT, criminal offences decreased, child protection cases decreased, and school attendance increased.
Katt et al. (2012) [59]	ON	Various rural and remote First Nations	N/A	Research, Intervention	A pilot study examining the feasibility and outcomes of a community-based Suboxone taper-to-low-dose-maintenance program for adults with prescription opioid dependence was conducted in a First Nation in Northern Ontario. The study determined the program is feasible and effective as an initial treatment, although abstinence is difficult to achieve and longer term maintenance may be required for some patients.
**Grey Literature**					
Centre for Addiction and Mental Health (2021) [2]	Canada	N/A	N/A	Report	The purpose of this document was to reach consensus regarding recommendations from existing clinical OAT guidelines. This document is comprised of guidelines blended with expert opinions and evidence-based literature. There is a need to collaborate with existing local services to prescribe OAT in a safe, accessible way (e.g. nurse practitioner, pharmacist). Telehealth expands the reach of OAT to people in rural communities. Buprenorphine improves retention and outcomes where access is limited.
Wells et al. (2019) [60]	Canada	N/A	N/A	Report	A literature search and a survey informed this environmental scan. Facilitators to timely access to OAT include walk-in style programming, transportation, increased staffing, lowered stigma, flexible appointment times, integrated treatment services, and the use of telehealth. Barriers to care include a lack of addiction services, stigma and judgment, long wait times, strict entry criteria, and a limited number of HCPs. Fee-for-service physicians may not want to risk no-show appointments (loss of income) and may view OAT patients as more complex and time-consuming patients to treat.
First Nations Health Authority (2020) [33]	BC	N/A	N/A	Fact sheet	Treatment options for OUD include different medical and psychosocial interventions to achieve spiritual, emotional, mental and physical healing and wellness. Health care providers must use a trauma-informed approach when working with clients who are experiencing substance use problems. BCCSU and UBC offer a free, self-directed online course “Provincial Opioid Addiction Treatment Support Program (POATSP)” that is recommended for HCP working in OUD care

**Fig. 1:**
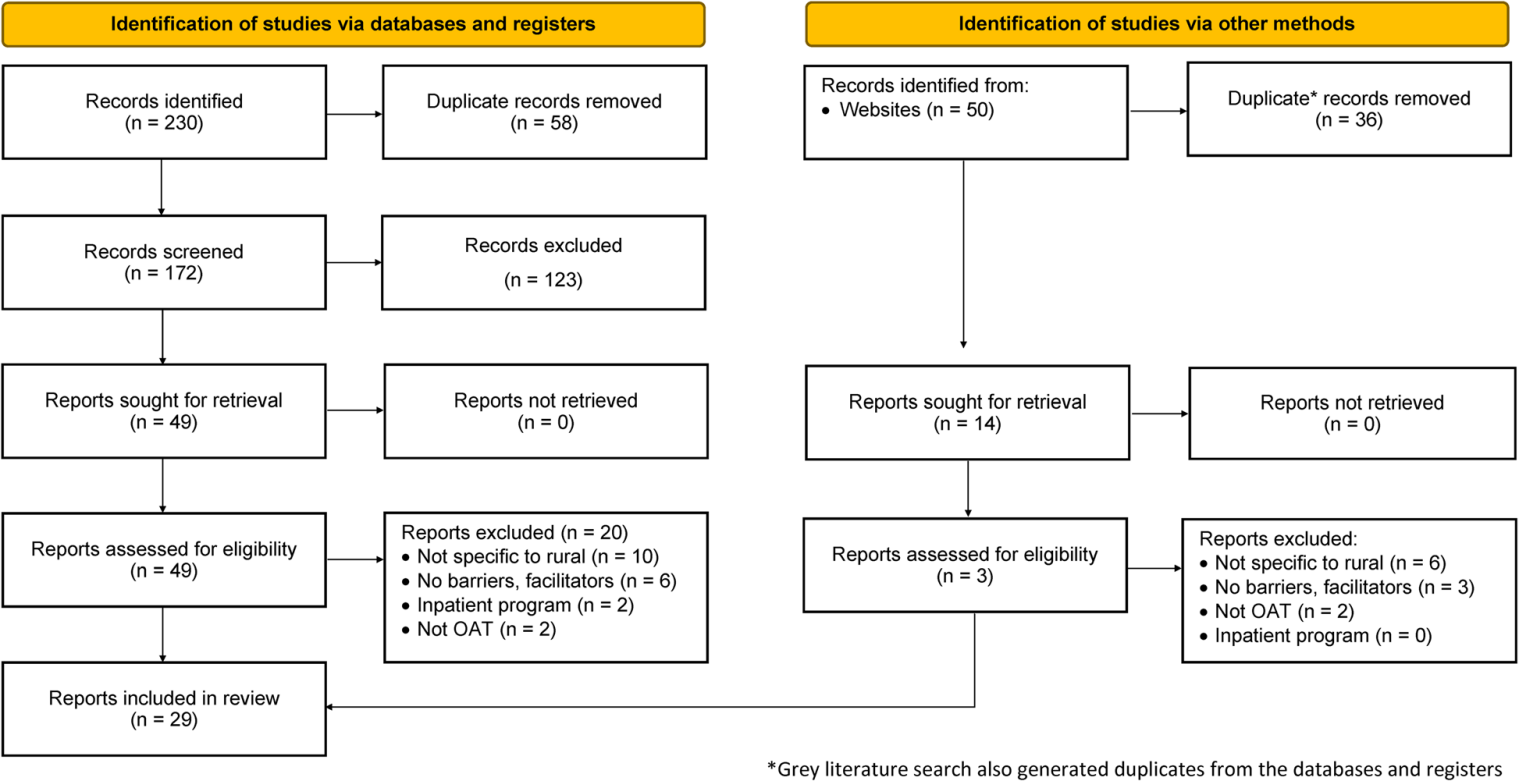
Selection criteria process for reviewed articles

The grey literature search strategy returned a total of 50 records from different websites (*n* = 11) and organizations (*n* = 6). Thirty-six (36) of the 50 records were duplicates from the databases and registers, which were not included in analysis but used to confirm the appropriateness of the search strategy. Fourteen items were included in the analysis (see Fig. 1). After screening titles and abstracts of executive summaries (where applicable), three full-text grey literature publications were included. The primary reasons for excluding items in this search were: lack of focus on rural, remote, and/or Indigenous settings (*n* = 6); lack of focus on barriers and facilitators of OAT (*n* = 3); and focus on an intervention other than outpatient OAT (*n* = 3).

### Results by type

Included documents are in Table 2 and organized by type.

#### Descriptive studies

Of the 14 included research studies, 11 were descriptive studies that reported on the barriers and facilitators to OAT in rural, remote, and Indigenous contexts in Canada [7, 19, 27, 29, 51–57]; ten of these studies were conducted in Ontario, with the remaining one in New Brunswick. Seven studies used administrative data, either alone or in combination with other methods (*n* = 2). Two studies were qualitative and two studies used a mixed method design. One study used a community-based participatory research design.

#### Intervention studies

Three articles described interventions to enhance access to and retention in OAT in rural, remote, and Indigenous contexts [6, 58, 59]. One intervention involved the use of telemedicine [58] and led to higher OAT retention rates. Kanate et al. [6] presented a compelling and promising community-developed program for First Nations communities that focused on traditional healing, substance use treatment, and substitution therapy. Katt et al. [59] discussed a community-based Suboxone taper to low dose maintenance program.

#### Commentaries

Nine of the articles were commentaries [41–49]. Six commentaries originated from work in Ontario, one from British Columbia, and two were general to Canada. Commentaries focused on the need to prioritize rural research (*n* = 1) and provide information about barriers, facilitators, and practice innovations (*n* = 8). It should be noted that the commentary by Webster [47] is essentially focused on the work of Katt et al. [59], a study that is described above. There was an overall emphasis on virtual care/telemedicine as a means to improve uptake and retention of OAT.

#### Other scholarly works

Other peer-reviewed literature included a case report [50], a narrative review [3], and a literature review [10].

#### Grey literature

The grey literature search strategy resulted in two reports and a fact sheet, all of which included rural, remote, and Indigenous considerations for OAT. The Centre for Addiction and Mental Health (CAMH) [2] recommended collaborating with existing services and using telehealth in partnership with local resources. Wells et al. [60] authored a report for the Canadian Agency for Drugs and Technologies in Health (CADTH), which highlighted the numerous barriers to care in rural settings and suggested ways to enhance care. The First Nations Health Authority (FNHA) [33] generated a fact sheet on pharmaceutical alternatives and OAT in Indigenous communities.

### Results by theme

Facilitators and barriers to outpatient OAT in rural and remote Canadian communities were organized into six themes: intrapersonal/patient factors; social/non-medical program factors; family/social context factors (including community factors); infrastructure/environmental factors; health care provider factors; and system/policy factors.

#### Intrapersonal/patient factors

Intrapersonal factors were frequently mentioned across all the reviewed documents. Barriers to patient success included polysubstance use, injection drug use, concurrent mental disorders, lack of awareness of services, apathy, fear of being judged or labeled, fear of child apprehension, fear of law enforcement, fear of disappointing others, maladaptive coping, and economic difficulties [3, 19, 53, 55]. Intrapersonal facilitators included a connection to spirituality and traditional beliefs as well as self-motivation [56]. Patient success was also fostered through: contingency planning; financial assistance for travel; access to stable and affordable housing; access to a community transportation system; incentives for attendance; access to formal substance use counseling; and logistical supports, such as assistance with health and identification cards, disability support applications, and appointments with child services workers/lawyers [2, 3, 19, 27, 41, 42, 56].

#### Social/non-medical programs

Social/non-medical services were considered important supports for those who were able to access them. In general, however, existing programs and services were described as disconnected, siloed, and lacking resources [48, 60]. As well, the COVID-19 pandemic limited or suspended traditional Indigenous healing practices that promoted success of OAT. OAT was enhanced by a range of medical and psychosocial interventions that aimed to achieve spiritual, emotional, mental, and physical healing and wellness [56]. Traditional Indigenous healing practices facilitated cultural connectedness and were identified as important aspects of recovery [48, 56]. In addition, group participation, social support, and peer encouragement were significant facilitators of OAT retention [42, 54, 56]. Researchers recommended the application of a local Indigenous worldview in the development and implementation of clinical, research, and program priorities in order to build strengths and increase local capacity. Researchers ultimately suggested that community members and PWUD must be directly involved in the development and delivery of programs to ensure they meet the needs of the community [41, 55].

#### Family/social context factors

Barriers to successful OAT initiation and treatment included the presence of domestic conflicts, stigma associated with OAT, and misunderstandings about OAT within the family unit [43, 54]. Due to crowded living conditions in many Indigenous communities, individuals often had privacy concerns regarding the use of telemedicine and virtual care. Patients who had a strong support system, including those with family members who were supportive of OAT and those who had support from a peer group, were more likely to succeed with OAT, especially when their family’s concerns were addressed in a timely fashion [55, 57].

Several community factors were also noted to help or hinder people on OAT. Barriers to effective access and treatment included public opposition to OAT/harm reduction strategies, stigma associated with OUD and OAT, community concerns about the safety of long-term OAT, and misconceptions about how OAT fits with spiritual beliefs [33, 52, 54, 60]. In addition, there were concerns about confidentiality and privacy within small towns and settlements. Several articles described how these barriers could be overcome, such as by openly addressing the community’s concerns through public discussions and education as well as by applying a local Indigenous worldview to the implementation of clinical care in First Nations communities, which can increase local capacity [55].

Researchers recommended creating community working groups to strengthen alliances between First Nations and provincial health services staff [43]. Community ownership of health programs generates buy-in, which translates into community support and better patient care [6, 46, 55]. Several authors underscored the necessity of helping communities understand that, since the opioid epidemic affects physical, mental, spiritual, and emotional well-being, community-wide healing strategies must address each of these elements [33, 46, 54]. Mamakwa et al. [55] described a particularly compelling model of care; while typical OAT programs are focused on the relationship between patient and HCP, the Sioux Lookout programs are a “community-wide welcoming back of addicted patients to their families and their previous roles” [55]. This program is cohort-based and induction into the program is a community celebration.

#### Infrastructure/environmental factors

Infrastructure and environmental factors, which are external to the patient but influence available services, were frequently mentioned across the literature. Barriers included the need to traverse significant geographical distances to access care, lack of transportation, extreme weather, seasonally accessible roads, lack of community services and resources, lack of local comprehensive treatment, under-resourcing of local services, and poor internet access (which limited access to telemedicine and virtual care) [3, 7, 27, 43, 48, 52, 56, 58]. These elements were particularly impacted by the COVID-19 pandemic, which limited patient care and patient monitoring, reduced access to withdrawal management, and disrupted all services and supports [29]. Facilitators to OAT included the availability of ancillary non-pharmacological substance use treatment and recovery services as well as multi-pharmacy approaches to OAT, which effectively increased access points, facilitated consent, and provided opportunities to help complex patients [7, 52].

#### Health care provider factors

A frequently cited factor that influenced patient care in rural, remote, and Indigenous communities was the general shortage of health care providers as well as the lack of providers (both nurse practitioners and physicians) who were trained and authorized to provide OAT [3, 10, 42, 44, 46, 49, 50, 55, 60]. The lack of legally authorized and qualified providers creates a bottlenecking of service entry, with long waitlists being identified as a predictor of attrition [55]. Even if a community has a qualified health care provider, access to OAT is dependent on many other external factors [41].

The current landscape is limited when it comes to renumeration models in substance use care and often providers are not compensated adequately for the time spent on complex issues they face when managing the health, trauma and social needs of those with substance use disorders [52]. Health care providers may face challenges in providing medication carries (take home doses) to these patients due to provincial regulations and even concerns about safe transport, storage, dispensing, diversion, and misuse of medications [48]. The literature highlighted a general shortage of physicians, nurses, addictions specialists, pharmacists, and pharmacies in rural areas, and noted that few providers were prepared to work with OAT patients. Rural pharmacies were often described as under-resourced, generally had very limited hours of operation, and did not routinely stock medications used in OAT [45, 47, 50, 52, 56]. Daily dispensing of OAT medications placed additional burdens on pharmacists and nursing stations, both of which are often closed on weekends [50, 52]. Finally, despite the staffing model outlined by Health Canada, Katt et al. [59] noted a distinct lack of long-term follow-up with patients in rural, remote, and Indigenous communities.

OAT was reported to be enhanced through: the use of a trauma-informed approach; higher doses of OAT to prevent withdrawal symptoms and discourage patients from self-medicating; unsupervised medication carries that provide pharmacists with greater flexibility in prescription deliveries; the use of telemedicine in partnership with local resources; education to increase the support and provide additional resources for physicians, nurse practitioners, and pharmacists to prescribe and manage OAT; strategies for safer home delivery of supplies; increased access to clinical mentorship opportunities for providers working with complex patients; and the provision of local, holistic, collaborative, and interdisciplinary care through the involvement of registered nurses, physicians, nurse practitioners, case managers, and addiction workers [2, 3, 27, 33, 48, 49, 52, 59]. The British Columbia Centre on Substance Use (BCCSU) and the University of British Columbia (UBC) offer a free, self-directed online course entitled “Provincial Opioid Addiction Treatment Support Program (POATSP)” that is recommended for health care providers involved in OAT [33]. Similarly, CAMH offers an online course. Patients also benefit from a long-term, harm reduction perspective (rather than an abstinence goal) as well as greater ease of accessibility and flexibility, including more accommodating appointment times and walk-in style programming [42, 60].

#### System/policy factors

The literature identified numerous bureaucratic and administrative barriers to OAT. For example, a comprehensive in-person evaluation of the patient must be completed before initiating OAT; this requirement is waived when telemedicine is used to compensate for distances and pandemic restrictions [49]. In addition, the strict entry criteria for OAT often presents a barrier to patients [60]. Furthermore, many physician compensation models are at odds with the complex needs of OAT patients [60]. Another challenge in the system is that daily witnessed dosing can be onerous for many rural and remote patients who must travel long distances [10, 60]. In some cases, take-home doses would require the patient to travel to a clinic on a daily basis for up to eight months, while Non-Insured Health Benefits only offers subsidized funding for four months of travel-related expenses [60]. As well, the requirement for nurse practitioners to obtain an exemption for prescribing opioids varies across the country, further reducing patient access. Lastly, Health Canada’s tendency to hire physicians on short-term contracts in Indigenous communities has contributed to opioid over-prescribing and subsequent crises in these communities [47]. Overall, the literature demonstrated that clinical practice guidelines for OAT failed to consider the realities of rural and remote practice. The literature suggested that regulatory changes were needed to enhance patients’ access to timely and appropriate comprehensive treatment [10, 56].

## Discussion

This integrative review revealed several facilitators and barriers, as well as strengths and gaps, with regards to OAT programs in rural and remote communities in Canada. Most of the literature about Canadian rural and remote OAT programs was based out of Ontario, with a dearth of literature on this topic from other provinces (BC, *n* = 2; NB, *n* = 1; NWT, *n* = 1; and none from the prairie provinces). The authors of this manuscript are aware of several innovative approaches to rural and remote OAT in provinces that have been unrepresented in the literature; it is unclear as to the source of the disconnect between practice innovation and scholarly dissemination.

In terms of study design, most studies (*n* = 14) were descriptive in nature, and many used administrative and electronic medical record (EMR) data. Three of the articles were intervention studies and three were qualitative studies. Commentaries (*n* = 9) and reviews (*n* = 3) comprised around half of the articles. The grey literature search strategy resulted in two reports and one fact sheet.

The themes across all of the documents bore resemblance to the social-ecological framework [61–63]. The social-ecological model of health posits that outcomes can be organized into five nested levels: intrapersonal factors, interpersonal factors, institutional factors, community factors, and public policy. This framework recognizes that the patient-provider dyad is inseparable from and impacted by infrastructure, policy, family, and community because all of the factors are networked rather than nested together [64]; each aspect has a different level of influence over other aspects.

**Fig. 2:**
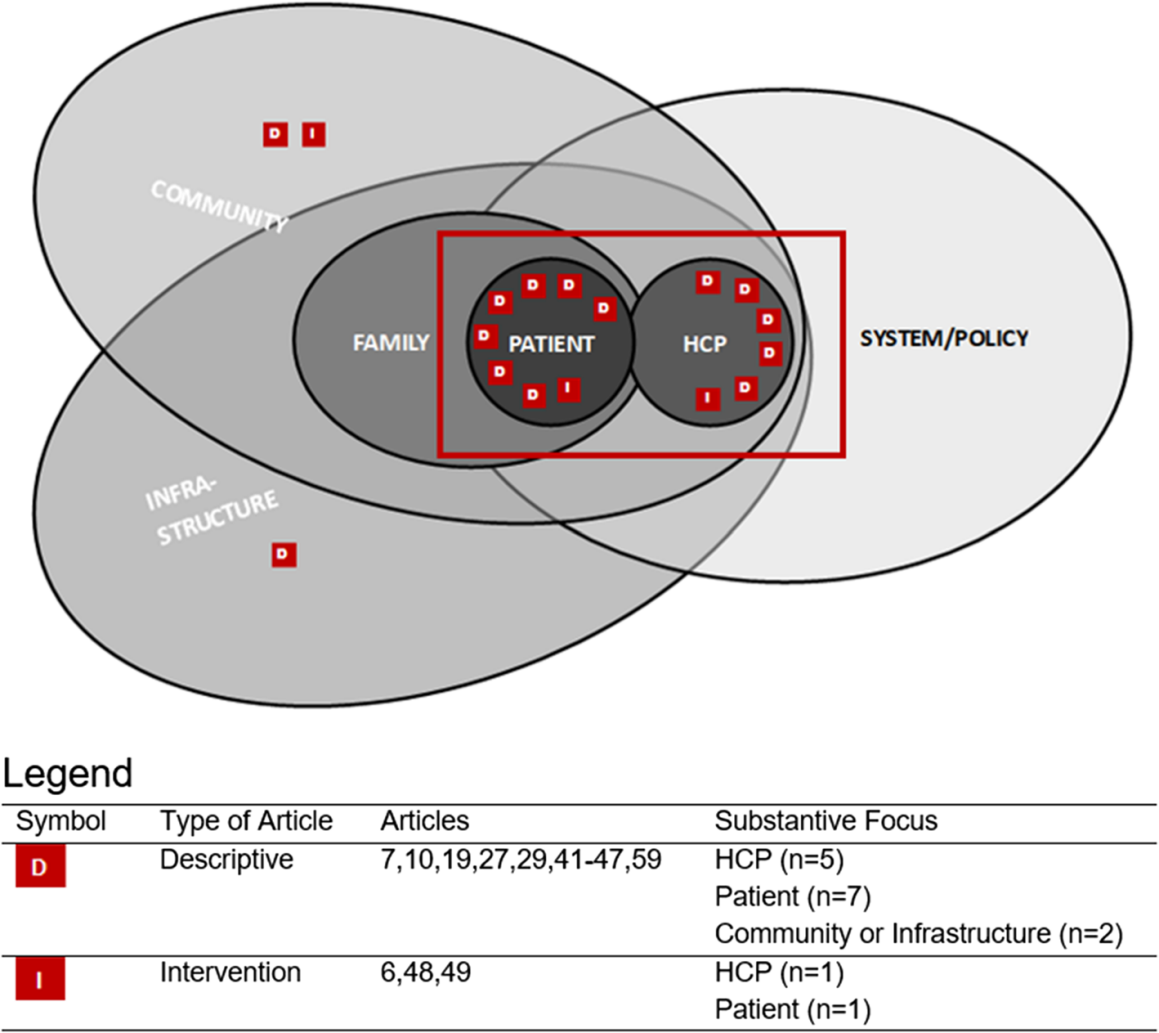
Primary or substantive focus of *research* on rural and remote OAT in Canada

That said, most of the literature (*n* = 12) focused on the patient-health care provider dyad (Fig. 2). As a result, our visual depiction of the extant literature considers patient and healthcare provider as distinct from each other and from the strict interpretation of “interpersonal factors.” Although the patient-provider dyad is prominently featured in the literature, it offers a narrow biomedical focus that is quite reductionistic when examined alongside more holistic approaches to OAT, as confirmed by the work of Mamakwa et al. [55]. Reductionistic perspectives are in direct conflict with the holism that typifies many Indigenous worldviews and approaches to care and, thus, may be a limiting factor in OAT success. Similarly, when reporting on a systematic review of OAT in American Indigenous communities, Mpofu et al. [65] describe the need for using multi-pronged interventions to manage OUD in these communities, establishing community-informed guidelines that are culturally appropriate ways, and capitalizing on community strengths that are rooted in traditions and culture.

Two of the most compelling studies [6, 55] in our review focused on community factors and highlighted a holistic approach to OAT care, nested within a community-based holistic model. Many authors suggested that programs be focused on a local Indigenous worldview and on strengthening local capacity. While most articles lacked sufficient detail on successful programs, Mamakwa et al. [55] described a community-centred and holistic model of OAT in which patients received connection and healing from their own community.

It is important that medical clinics not limit patient engagement and success to factors within the patient-provider dyad, as this will overlook the importance of community support, culture, social restoration, and belonging. Cultural awareness and creative and adaptive strategies are needed to respond to the geographical, cultural, and institutional circumstances that typify rural and remote communities [65, 66]. Although evaluation of OAT programs is typically centred on the patient-provider dyad, the Sioux Lookout OAT program is community-wide and focused on welcoming substance-dependent patients back to their families and previous roles. The Sioux Lookout program is cohort-based; induction into the program is revered as a community event whereby local leaders, friends, and relatives support the inductees. In addition, psychosocial treatment programs can provide culturally appropriate meeting places for patients to gather in healing circles and other traditional events. Research from the US also describes the need for holistic and culturally relevant care that is rooted in the local community with its history, traditions and support networks; authors agree that the opioid epidemic must be addressed at both the individual and community levels and address health policy factors that contribute to the opioid epidemic [31, 67, 68].

This review raises several key issues. First, policymakers and medical infrastructure must become catalysts for change to ensure OAT is more accessible to people in resource-poor rural and remote areas. Policy changes and evaluation research are needed to support innovative practices in rural and remote areas. This review is critical to the advancement of a targeted research and policy agenda to drive the provision of innovative and effective health services [69]. In the US, Johnson et al. [70] report that federal regulations and local infrastructure exacerbates the transportation barrier and is a deterrent to care in rural communities. The US, like Canada, also structures rural and remote physician services under federal provision, which underscores the need for increased governmental support of health care in these settings [71].

Second, Indigenous-led participatory action research is required to investigate successful OAT programs. The success of these programs may be attributed to the fact that they are designed, led, and implemented by the community. In addition, the delivery of these Indigenous-led programs differs from that of non-Indigenous/Western programs, which are inherently reductionist. Community-based approaches require further investigation and broader implementation. Intervention research is also needed.

Third, the evaluation of community impacts is another important area of future research that aligns with a social-ecological perspective. Community-based programs were associated with high retention rates and positive community-wide results (i.e., lower crime rates, fewer child protection cases, higher school attendance rates, and fewer drug-related medical evacuations to hospital). By monitoring community-level data and linking it to substance use statistics (including treatment and OAT), important relationships can be explored and leveraged into action.

## Conclusion

The COVID-19 pandemic has exposed gaps and weaknesses in existing systems, particularly within health care in rural and remote communities. Thus, the pandemic has exacerbated existing crises in communities that are already vulnerable; urgent action is desperately needed to reverse these dire trends. This integrative review demonstrated that while most OAT studies in Canada have focused on the patient-provider dyad as the locus of patient success, such a reductionistic perspective may be contributing to the problem. Rather, interventions that are holistic, deeply situated in the community, culturally embedded and rooted in traditional healing, may hold greater promise for helping individuals, families and communities to heal and experience restoration. Even if the path forward is unclear, there are powerful examples of Indigenous-led and community-based approaches to address the opioid crisis in rural and remote settings. These exemplars entreat further exploration, implementation and evaluation.

## Data Availability

Not applicable.

## Abbreviations

HCP: Health care provider
OAT: Opioid agonist therapy
OUD: Opioid use disorder
PWUD: People who use drugs
